# Mesenchymal stromal cells from myelodysplastic and acute myeloid leukemia patients display in vitro reduced proliferative potential and similar capacity to support leukemia cell survival

**DOI:** 10.1186/s13287-018-1013-z

**Published:** 2018-10-25

**Authors:** Giulia Corradi, Carmen Baldazzi, Darina Očadlíková, Giovanni Marconi, Sarah Parisi, Nicoletta Testoni, Carlo Finelli, Michele Cavo, Antonio Curti, Marilena Ciciarello

**Affiliations:** Department of Experimental, Diagnostic and Specialty Medicine, Institute of Hematology “L. & A. Seràgnoli”, University of Bologna, Azienda Ospedaliero—Universitaria Policlinico S. Orsola-Malpighi Bologna, Via Massarenti 9, 40138 Bologna, Italy

**Keywords:** Mesenchymal stromal cells, Acute myeloid leukemia, Myelodysplastic syndrome, Leukemic microenvironment

## Abstract

**Background:**

Mesenchymal stromal cells (MSCs) are an essential element of the bone marrow (BM) microenvironment, playing a crucial function in regulating hematopoietic stem cell proliferation and differentiation. Recent findings have outlined a putative role for MSCs in hematological malignancy development. So far, conflicting results have been collected concerning MSC abnormalities in acute myeloid leukemia (AML) and myelodysplastic syndrome (MDS). In particular, a considerable amount of evidence has been accumulated strongly supporting a permissive role of MSCs in malignancy evolution to MDS, while a potentially causative or promoting function performed by MSCs in AML has not yet been fully clarified. Here, we compared MSCs isolated from healthy, MDS, and AML subjects to investigate MSC alterations and to emphasize putative common and/or diverse features.

**Methods:**

We isolated and expanded MSCs from AML patients (AML-MSCs) and MDS patients (MDS-MSCs), and we analyzed and compared their phenotypic and functional properties with respect to each other and versus healthy donor-derived MSCs (HD-MSCs).

**Results:**

We found that stable MSC cultures could be easily established from HD and MDS mononuclear BM-derived cells, while a substantial fraction (25%) of AML patients failed to yield MSCs. Nevertheless, isolated MDS-MSCs and AML-MSCs, as well as HD-MSCs, contained the basic features of MSCs. Indeed, they displayed similar surface marker expression and efficient capacity to differentiate versus osteogenic and adipogenic lineage in vitro. We also proved that MDS-MSCs and AML-MSCs, analyzed by fluorescence in-situ hybridization, did not harbor leukemic cell cytogenetic abnormalities. Moreover, MDS-MSCs and AML-MSCs were similar in terms of ability to sustain AML cell viability and immune-regulatory capacity. However, we were also able to detect some differences between AML-MSCs and MDS-MSCs. Indeed, we found that the frequency of rescued MSCs was lower in the AML group than in the HD and MDS groups, suggesting that a reduced number of MSC precursors could inhabit AML BM. Instead, MDS-MSCs showed the lowest proliferative capacity, reflecting some intrinsic and particular defect.

**Conclusions:**

Overall, our results elucidated that MDS-MSCs and AML-MSCs did not show macroscopic and/or tumor-related defects, but both displayed functional features potentially contributing to favor a leukemia-protective milieu.

## Background

Myelodysplastic syndrome (MDS) and acute myeloid leukemia (AML) are two biologically and genetically heterogeneous groups of clonal myeloid neoplastic disorders characterized by hematopoietic stem cell (HSC) dysregulation and ineffective hematopoiesis [1, 2]. In particular, MDS carries a substantial risk of progressing into AML, although the molecular mechanism underlying this transformation remains unknown. Neoplastic cells derived from the majority of MDS and AML patients harbor cytogenetic and molecular abnormalities thought to account for the outgrowth and differentiation defects of these cells and ultimately for disease pathogenesis [1, 2]. However, recent evidence implies that the pathogenesis of these and other hematological malignancies depends not only on cell intrinsic factors, but is also supported by the bone marrow (BM) microenvironment in general, and specifically by mesenchymal stromal cells (MSCs). MSCs provide a substantial contribution to the creation of a hematopoietic niche [3, 4], and play an essential role in normal hematopoiesis by regulating HSC proliferation and differentiation. In the last few years, it has been demonstrated that specific changes in MSCs can initiate leukemia in mice [5–7]. This evidence in murine models has provided the rationale to explore the biological and functional features of human MSCs in hematological patients. It was consequently shown that, in various hematopoietic disorders, MSCs presented alterations in the expression of cell adhesion molecules and cytokines, and had an impaired immunosuppressive efficiency and/or a reduced capacity to proliferate or to support hematopoiesis [8–14]. However, so far, data regarding MSC alterations and their contribution to AML and MDS disease mechanisms and/or treatment outcomes have been controversial. In particular, the characterization of AML patient-derived MSCs has been poor and not conclusive, likely due to the high level of disease heterogeneity and to the limited cohort of analyzed patients [15–17]. Although two studies have recently characterized a more robust cohort of MSCs isolated from AML patients, even establishing a link between MSC alterations and treatment outcome [18, 19], a potentially causative or supporting role of MSCs in AML has still not been adequately explored [20]. On the contrary, evidence has accumulated strongly arguing in favor of an MSC contribution to disease pathogenesis in MDS [21]. Indeed, BM samples derived from MDS patients are very challenging to engraft in murine models or fail to confer their MDS clinical phenotype [22, 23], suggesting a permissive role of the microenvironment in MDS. In this study, we investigated and compared MSCs isolated from healthy donors, MDS subjects, and AML subjects in terms of different biological parameters with the aim of highlighting phenotypic and functional alterations and shared features.

## Methods

### Patients and healthy controls

BM samples were obtained from 32 AML patients (18 males, 14 females; median age 60.5 years) and 26 MDS patients (17 males, 9 females; median age 78 years). In addition, BM from 12 healthy donors (HDs) (9 males, 3 females; median age 42 years) was used as the control (see Table 1).

**Table 1:** Biological, cytogenetic, and molecular characteristics of HD, MDS, and AML patients

Patient ID	Cytogenetics	Molecular	Gender	Age (years)	Risk	MSCs/MNCs	Cytogenetic alteration in MSCs
AML01	46,XX,t(6;9) (p22;q34)[20]^a^	NPMwt FLT3-ITD	Female	45	High^b^	0.97	ND
AML02	46,XY[20]	ND	Male	80	Intermediate	ND	ND
AML03	46,XY[20]	NPMmut FLT3wt	Male	63	Low	1.5	ND
AML04	46,XY,inv(16) (p13q22)[20]	NPMwt FLT3wt TP53wt	Male	55	Low	0.1	Negative
AML05	46,XX[20]	NPMmut. FLT3wt TP53wt	Female	63	Low	ND	ND
*AML06* ^c^	*ND*	*NPMmut* *FLT3-ITD TP53wt*	*Male*	*70*	*Intermediate*	*ND*	*ND*
AML07	46,XY,t(15;17) (q22;q22)[20]	ND	Male	39	NA	0.2	Negative
AML08	46,XX[20]	NPMmut FLT3-ITD/TKD	Female	47	Intermediate	1.3	ND
AML09	46,XX[20]	NPMmut FLT3-ITD	Female	61	Intermediate	0.7	ND
*AML10*	*47,XY,+ 8[20]*	*NPMwt FLT3wt*	*Male*	*68*	*Intermediate*	*ND*	*ND*
*AML11*	*46,XY[20]*	*NPMmut* *FLT3-ITD TP53wt*	*Male*	*73*	*Intermediate*	*ND*	*ND*
AML12	47,XY,+der(3) del(3)(p11), t(10;11;19) (p12;q23;q13)[20]	NPMwt FLT3wt	Male	17	High	ND	Negative
AML13	44,XX,+der(3) t(3;20)(p12;p11),del(5)(q13q33), −7,–13,t(13;20) (q12;p11), −17,der(21) t(17;21) (q11;q22), +mar[14]/ 45,XX,t(1;16) (q12;q11),del(5) (q13q33),del(6) (q21q25), − 7,add(22)(q13)[6]	NPMwt FLT3wt TP53mut	Female	60	High	0.3	ND
AML14	46,XX,t(16;16) (p13;q22)[20]	NPMwt. FLT3wt TP53wt	Female	46	Low	ND	ND
AML15	46,XY,t(6;11) (q27;q23)[20]	NPMwt FLT3wt TP53wt	Male	19	High	5.6	ND
AML16	46,XY,inv(16) (p13q22)[18]/ 47,XY,inv(16) (p13q22),+ 22[2]	NPMwt FLT3wt TP53wt	Male	26	Low	ND	ND
AML17	46,XX,t(9;11) (p22;q23) [20]	NPMwt FLT3wt	Female	55	Intermediate	0.1	ND
*AML18*	*47,XX,+X,* *add(7)(q34)[19]/* *47,XX,+X[1]*	*ND*	*Female*	*72*	*Intermediate*	*ND*	*ND*
AML19	46,XX[20]	FLT3-ITD	Female	61	High	6.9	ND
AML20	46,XX[20]	FLT3-ITD	Female	73	High	0.5	ND
*AML21*	*47,XX,del(5)* *(q22q33),+ 8[20]*	*ND*	*Female*	*62*	*High*	*ND*	*ND*
AML22	46,XY[20]	FLT3-ITD	Male	79	High	6.4	ND
AML23	46,XX[20]	NPMmut FLT3-TKD	Female	76	Low	5.6	ND
AML24	46,XX,der(4) t(1;4)(q32;q31) [20]	NPMwt FLT3wt TP53wt	Female	55	Intermediate	3.7	ND
*AML25*	*46,XY[20]*	*NPMwt FLT3-ITD TP53wt*	*Male*	*31*	*Intermediate*	*ND*	*ND*
AML26	ND	NPMwt FLT3wt TP53wt	Male	57	NA	0.5	ND
*AML27*	*ND*	*NPMmut* *FLT3wt* *TP53wt*	*Male*	*59*	*Low*	*ND*	*ND*
AML28	46,XY,–7,+ 21[20]	NPMwt FLT3wt TP53wt	Male	74	High	ND	ND
AML29	46,XX[20]	NPMwt FLT3wt TP53wt	Male	69	Intermediate	ND	ND
AML30	47,XY,inv(16) (p13q22),+ 8[14]/46,XY,inv.(16) (p13q2)[6]	NPMwt FLT3wt TP53wt	Male	56	Low	ND	ND
AML31	46,XY,t(6;9) (p22;q34)[20]	NPMwt FLT3-ITD	Male	75	Intermediate	3.2	ND
*AML32*	*ND*	*ND*	*Female*	*71*	*ND*	*ND*	*ND*
MDS1	46,XX[20]		Female	79	Low/intermediate^d^	0.8	ND
MDS3	46,XY[20]		Male	62	Int2/High	ND	ND
MDS4	46,XY[20]		Male	78	Int1/Low	0.5	ND
MDS5	46,XY[20]		Male	86	Low/Low	3.3	ND
MDS6	46,XX[20]		Female	61	High/High	2.7	ND
MDS9	46,XY[20]		Male	79	Low/Low	1.2	ND
*MDS11*	*46,XY,del(20)* *(q11q13)[20]*		*Male*	*53*	*Int1/intermediate*	*ND*	*ND*
MDS12	71–74,XXX, +der(2)t(2;17) (p11;p12), del(5)(q13q33), +der(5)del(5) (q13q33), −7[5]/46,XX[5]		Female	73	High/high	6.7	Negative
MDS15	46,XX,del(5) (q13q33)[5]/ 46,XX, del(5)(q13q33), del(11)(q21q25) [3]		Female	76	Int1/Low	5.6	ND
MDS16	46,XY[20]		Male	80	Low/low	2.6	ND
MDS17	46,XY[20]		Male	68	High/very high	6	ND
MDS18	46,XY[20]		Male	66	Low/low	8.3	ND
MDS21	46,XY[20]		Male	78	Low/low	ND	ND
MDS22	46,XY[30]^e^		Male	61	Low/low	4.4	ND
MDS24	46,XX[20]		Female	83	Low/low	2.5	ND
*MDS25*	*46,XX,del(20)* *(q11q13)[20]*		*Female*	*81*	*Low/low*	*ND*	*ND*
MDS26	46,XY[20]		Male	54	Low/low	8.9	ND
MDS32	46,XX[20]		Female	67	Low/low	2.5	ND
MDS33	ND		Male	76	Int1/high	3.6	ND
MDS34	46,XY[20]		Male	90	Int1/low	3.5	ND
MDS35	46,XX[20]		Female	83	Low/low	2.5	ND
MDS36	47,XY,+ 8[20]		Male	82	Int2/very high	1.8	ND
MDS38	46,X,idic(X) (q13)[16]/45,X, –X[2]/47,X, del(X)(q13), +idic(X)(q13) [1]/47,del(X) (q13),idic(X) (q13) + idic(X) (q13)[1]		Female	86	Int1/low	1.1	ND
MDS39	46,XY[20]		Male	87	Int1/intermediate	*ND*	*ND*
MDS43	49,XY,+ 1,del(5) (q13q33), +der(5)del(5) (q13q33), + 11[18]/47XY, del(5)(q13q33), +der(5)del(5) (q13q33)[1]/ 46XY,del(5) (q13q33)[1]		Male	79	Int2/high	ND	Negative
MDS45	46,XY,del(13) (q12q14)[3]/ 46,XY[17]		Male	65	High/very high	ND	Negative
HD01	46,XY	–	Male	22	–	ND	ND
HD02	46,XY	–	Male	19	–	ND	ND
HD03	46,XY	–	Male	43	–	8.4	ND
HD04	46,XY	–	Male	44	–	2.2	ND
HD05	46,XY	–	Male	32	–	5.4	ND
HD06	46,XY	–	Male	41	–	2	ND
HD07	46,XX	–	Female	62	–	0.5	ND
HD08	46,XY	–	Male	60	–	14.3	ND
HD09	46,XX	–	Female	43	–	9.4	ND
HD10	46,XX	–	Female	52	–	7	ND
HD11	46,XY	–	Male	22	–	11.2	ND
HD12	46,XY	–	Male	38	–	ND	ND

*AML* acute myeloid leukemia, *HD* healthy donor, *MDS* myelodysplastic syndrome, *MNC* mononuclear seeded cell, *MSC* mesenchymal stromal cell, *ND* not determined, *int1* intermediate 1 risk, *int2* intermediate 2 risk

^a^Karyotypes described according to the International System for Human Cytogenetic Nomenclature (ISCN 2016) [53, 54]

^b^Risk evaluation following ELN 2017 [55]

^c^Patients in which MSCs were not isolated are indicated in italics

^d^Risk evaluation following IPSS/IPSS-R [56, 57]

^e^CEP 8 spectrum orange DNA probe shows 3 signals in 4% of interphase nuclei

### MSC isolation and culture

BM-derived MSCs were isolated from BM aspirates of HDs (HD-MSCs) or patients affected by acute myeloid leukemia (AML-MSCs) or myelodysplastic syndrome (MDS-MSCs) at diagnosis, and were expanded ex vivo as previously described [24]. Briefly, the mononuclear cell (MNC) fraction was separated by centrifugation over a Ficoll-Paque gradient (Lympholyte CL5020 1.077 g/ml; Cedarlane), resuspended in proliferation medium consisting of low-glucose Dulbecco’s modified Eagle’s medium (DMEM; Lonza), 10% fetal bovine serum (FBS; Thermo Fisher Scientific), 2 mM l-glutamine, and 1% penicillin/streptomycin (pen/strep) (MP Biomedicals), and plated at an initial seeding density of 1.6 × 10^5^ cells/cm^2^. After 2–3 days, the nonadherent cell fraction was removed by rinsing cells with phosphate-buffered saline solution (PBS), and monolayers of adherent cells were cultured until they reached 70–80% confluence. Cells were then detached by trypsin solution (0.25% trypsin/0.1% EDTA in PBS w/o calcium w/o magnesium w/ Phenol Red) (Aurogene, Rome, Italy), reseeded at a density of 3.5 × 10^3^ cells/cm^2^, and used for experiments within passages 3–5. Cell growth was analyzed by direct cell counts at each passage.

### Immunophenotype

For immunophenotype studies, dual-color immunofluorescence was performed using the following panel of phycoerythrin (PE)-conjugated or fluorescein isothiocyanate (FITC)-conjugated monoclonal antibodies: anti-human CD13, anti-human CD19, anti-human CD34, anti-human HLA-DR, anti-human CD44, anti-human CD45, anti-human CD73 (Becton Dickinson), anti-human CD14, anti-human CD29, anti-human CD105 (Biolegend), and anti-human CD90 (Chemicon). The cell autofluorescence level was used as the negative control. For cell-surface staining, 1 × 10^5^ cells were incubated, in the presence of the antibodies listed, in PBS/0.5% FBS at room temperature with light protection for 15 min. Cells were rinsed in PBS and analyzed by flow cytometry (FACScanto II equipment; Becton Dickinson). A minimum of 10,000 events was collected in list mode on FACSDiva software.

### Differentiation potential

To induce osteogenic differentiation, MSCs were seeded at 3.1 × 10^3^ cells/cm^2^ and grown in osteogenic differentiation medium (Lonza) containing l-glutamine, mesenchymal cell growth serum MCGS, dexamethasone, ascorbate, β-glycerophosphate, and pen/strep. The medium was replaced every 3–4 days. Cell cultures were stopped at day 21 for histological staining and total RNA extraction. Calcium deposition was determined using Alizarin red staining as previously described [24]. Briefly, cells were fixed in 10% paraformaldehyde (PFA) in PBS at room temperature for 15 min, and rinsed with PBS and distilled water. Fixed cultures were stained with 40 mM Alizarin red solution (Sigma Aldrich), pH 4.2, with gentle shaking at room temperature for 75 min and rinsed with distilled water. To induce adipogenic differentiation, MSCs were seeded at 2.1 × 10^4^ cells/cm^2^ on a Lab-Tek II coverglass chamber (Nalge-Nunc) and grown for 3 days in adipogenic induction medium (Lonza) containing additional h-insulin, l-glutamine, MCGS, dexamethasone, indomethacin, 3-isobuty-1-methyl-xanthine, and pen/strep followed by 3 days in adipogenic maintenance medium containing h-insulin, l-glutamine, MCGS, and pen/strep. Both steps were repeated up to day 18 when cell cultures were stopped for histological staining and total RNA extraction. Fat droplets within adipogenic differentiated cells were identified using the Oil Red O staining method as previously described [24]. Briefly, cells were fixed in 10% PFA in PBS at room temperature for 1 h and rinsed in 60% isopropanol. The isopropanol was removed and the wells were completely dried and stained with 0.6% (*w*/*v*) Oil Red O solution (Sigma Aldrich) with gentle shaking at room temperature for 15 min. Images were collected with an Axiovert 40 CFL microscope (Carl Zeiss Microscopy).

### Total RNA extraction, reverse transcription, and quantitative real-time polymerase chain reaction

Total RNA was isolated using an RNeasy Micro Kit (Qiagen) according to the manufacturer’s instructions and quantified by NanoDrop ND-1000 spectrophotometer (NanoDrop Technologies). For cDNA synthesis, 1 μg of denatured total RNA was reverse transcribed using an Improm II kit (Promega) and random hexamers (Promega) in a 20 μl final volume according to the manufacturer’s instruction. Quantitative real-time polymerase chain reaction (qRT-PCR) was performed using the ABI-PRISM 7900 Sequence Detection System (Applied Biosystems). The qRT-PCR reactions were performed using a 96-well Optical Reaction Plate. For each PCR run, 1 μl of cDNA product was mixed with 2× Platinum Super mix (Thermo Fisher Scientific) in a total volume of 25 μl. The thermal cycling conditions consisted of an initial stage of 50 °C for 2 min, and 95 °C for 10 min, 40 cycles of melting at 95 °C for 15 s, and annealing and elongation at 60 °C for 1 min. Threshold cycle (C_t_) values for differentiation specific genes (i.e., peroxisome proliferator activated receptor gamma (PPARγ), Runt-related transcription factor (RUNX) 2) and an endogenous reference gene (i.e., glyceraldehyde 3-phosphate dehydrogenase (GAPDH)) were determined automatically using the 7900 ABI PRISM system (Applied Biosystems). Relative quantification was calculated using the ΔC_t_ comparative method [25]. Briefly, the relative level of a specific cDNA was calculated by subtracting the C_t_ value of the endogenous reference gene from the C_t_ value of the specific gene. PPARγ or RUNX2 cDNA levels in undifferentiated cells were taken as 1. In some experiments, cDNA synthetized as already described from a Universal RNA (Agilent genomics) was used as the reference value and taken as 1. All reactions were performed in duplicate. Primer probes for PPARγ, Hs01115513_m1, RUNX2, Hs00231692_m1, GAPDH, and Hs00266705_g1 were purchased from Applied Biosystems.

### Fluorescent in-situ hybridization

Molecular cytogenetic analysis was performed on BM-derived AML or MDS cells and BM-derived MSCs isolated as already described. All cells were treated with hypotonic solution and fixed with methanol–acetic acid solution (3:1). Fluorescent in-situ hybridization (FISH) was carried out on fixed cells according to the manufacturer’s instructions with appropriate specific probes. The following commercially avaialble probes were used for MSC analysis: EGR1 FISH Probe Kit, LSI MLL Dual Colour, Break Apart Rearrangements Probe and LSI PML/RARα Dual Colour, and Dual Fusion Translocation Probe Kit (Vysis); and CBFβ/MYH11 Translocation, Dual Fusion Probe, and RB1 Deletion Probe (Cytocell). Images were analyzed using a fluorescence microscope NIKON E1000 equipped with FITC/TRITC/AQUA/DAPI filter sets and the Genikon imaging system software (Nikon Instruments). At least 200 nuclei were counted for each sample.

### Coculture experiments

MSCs were seeded at the density of 20,000 cells/cm^2^ and after 24 h AML cells were seeded with transwells on MSC layers (1:10). After 4 days of cocultures, AML cells were harvested and analyzed by flow cytometry. In apoptosis experiments, after 4 days of cocultures, AML cells were harvested and labeled with annexin-V/propidium iodide (PI) (annexin-V-FLUOS-kit; Roche). Briefly, cells were washed in PBS and then incubated with Annexin-V-FLUOS/PI incubation buffer with light protection at room temperature for 15 min and analyzed by flow cytometry as already described.

In proliferation experiments, before seeding, AML cells were labeled with carboxyfluorescein succinimidyl ester (CFSE) (BioLegend). Briefly, cells were washed twice in PBS and incubated with CFSE (5 μM) with light protection at room temperature for 4 min. Cells were then washed twice in cold medium (RPMI, Lonza) and analyzed by flow cytometry as already described.

### In-vitro Treg induction

MSCs derived from HDs, AML patients, or MDS patients were cocultured for 7 days in autologous RPMI with allogeneic peripheral blood mononuclear cells (PBMCs) (ratio 10:1). After 7 days, PBMCs were harvested and stained using the intracellular staining kit FOXP-3/Transcription Factor Buffer Set (eBioscience/Thermo Fisher Scientific) including the monoclonal antibodies (mAbs) PE-conjugated anti-human FOXP3 (clone PCH101; Thermo Fisher Scientific), APC-H7-conjugated CD3 (clone SK7; BD/Pharmingen), FITC-conjugated CD4 (clone RPA-T4; Thermo Fisher Scientific), and APC-conjugated CD25 (clone BC96; eBioscience/Thermo Fisher Scientific). For each sample, isotype-matched irrelevant mAb staining was used as the control. At least 10,000 events were analyzed by flow cytometry as already described. FoxP3^+^/CD4^+^/CD25^+^ cells were gated on CD4^+^ cells.

### Data analysis

Data are presented as mean ± SEM of at least three independent determinations. Statistical differences between groups were determined by Student’s *t* test or one-way analysis of variance (ANOVA) followed by Bonferroni’s post-hoc test for multiple comparison. All analyses were performed using GraphPad Prism software (version 6.0). Differences were considered statistically significant at *p* < 0.05.

## Results

### MDS-MSCs and AML-MSCs show a reduced proliferative capacity

**Fig. 1:**
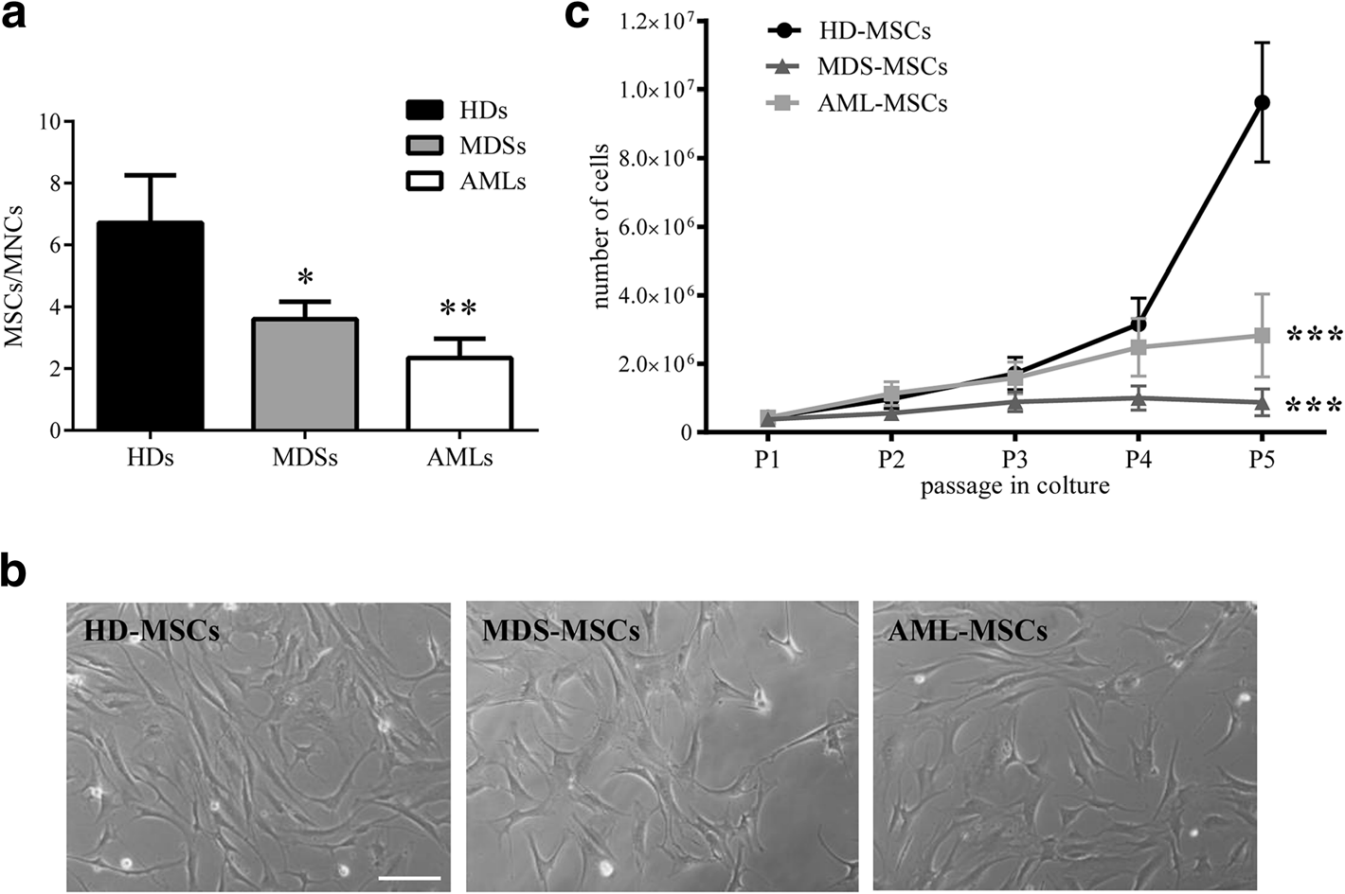
MSCs derived from MDS and AML patients show normal morphology but reduced proliferative capacity. **a** MSCs isolated at P1 normalized to number of BM-derived mononuclear cells seeded in three analyzed groups. Results expressed as mean ± SEM calculated from data obtained from independent samples of HDs (*N* = 9), MDS patients (*N* = 19), AML patients (*N* = 16) (*P < 0.05; **P < 0.01). **b** Representative field of exponential growing culture of HD-MSCs, MDS-MSCs, and AML-MSCs. Magnification 10×; scale bar, 100 μm. **c** Comparison of cell counts in BM-derived HD-MSCs, MDS-MSCs, and AML-MSCs at each passage. Results expressed as mean ± SEM calculated from data obtained from at least seven independent samples (***P < 0.001 at P5). MSC mesenchymal stromal cell, MNC mononuclear cell, HD-MSC mesenchymal stromal cell from healthy donor, MDS-MSC mesenchymal stromal cell from myelodysplastic syndrome patient, AML-MSC mesenchymal stromal cell from acute myeloid leukemia patient, P cell culture passage

We isolated and expanded MDS-MSCs (*N* = 26) and AML-MSCs (*N* = 32) at diagnosis from treatment-free patients to rule out potential bias due to chemotherapy effects. As a control, MSCs were isolated and expanded from healthy subjects (HD-MSCs, *N* = 12). MSCs were successfully obtained from all samples of HDs and from almost all the MDSs (88.5%, 23 out of 26), while only 75% (24 out of 32) of the AML samples were able to generate MSCs (Table 1). Furthermore, as shown in Fig. 1a, we calculated the number of MSCs isolated after the first passage (P1) normalized to the number of BM-isolated mononuclear seeded cells (MNCs). Although there was, as expected, some variability within each group, we found that this ratio (i.e., the frequency of rescued MSCs) was significantly lower in the AML group than in the HD group (*P* < 0.01), while the MDS-MSC frequency had an intermediate value (*P* < 0.05 vs HD; not significant vs AML) (Fig. 1a). This suggested that a reduced number of MSC precursors may inhabit the BM of MDS and AML patients. Despite the differences in the isolation efficiency, MSCs were all plastic adherent and showed a typical fibroblastoid elongated shape with no obvious differences between groups (Fig. 1b). In order to compare proliferative capacity, MSCs isolated from the three groups were plated and cultured in the same conditions until confluence. Direct cell counts were determined at each passage from P1 to P5. We observed that the number of HD-MSCs consistently increased at each passage while the number of MDS-MSCs and AML-MSCs slightly rose at the first passage and then remained more or less unchanged (*P* < 0.001 at P5). This pattern was particularly exacerbated in the MDS-MSCs (Fig. 1c). These data suggested that AML-MSCs and especially MDS-MSCs presented some intrinsic growing defect.

### MDS-MSCs and AML-MSCs show typical MSC features

**Fig. 2:**
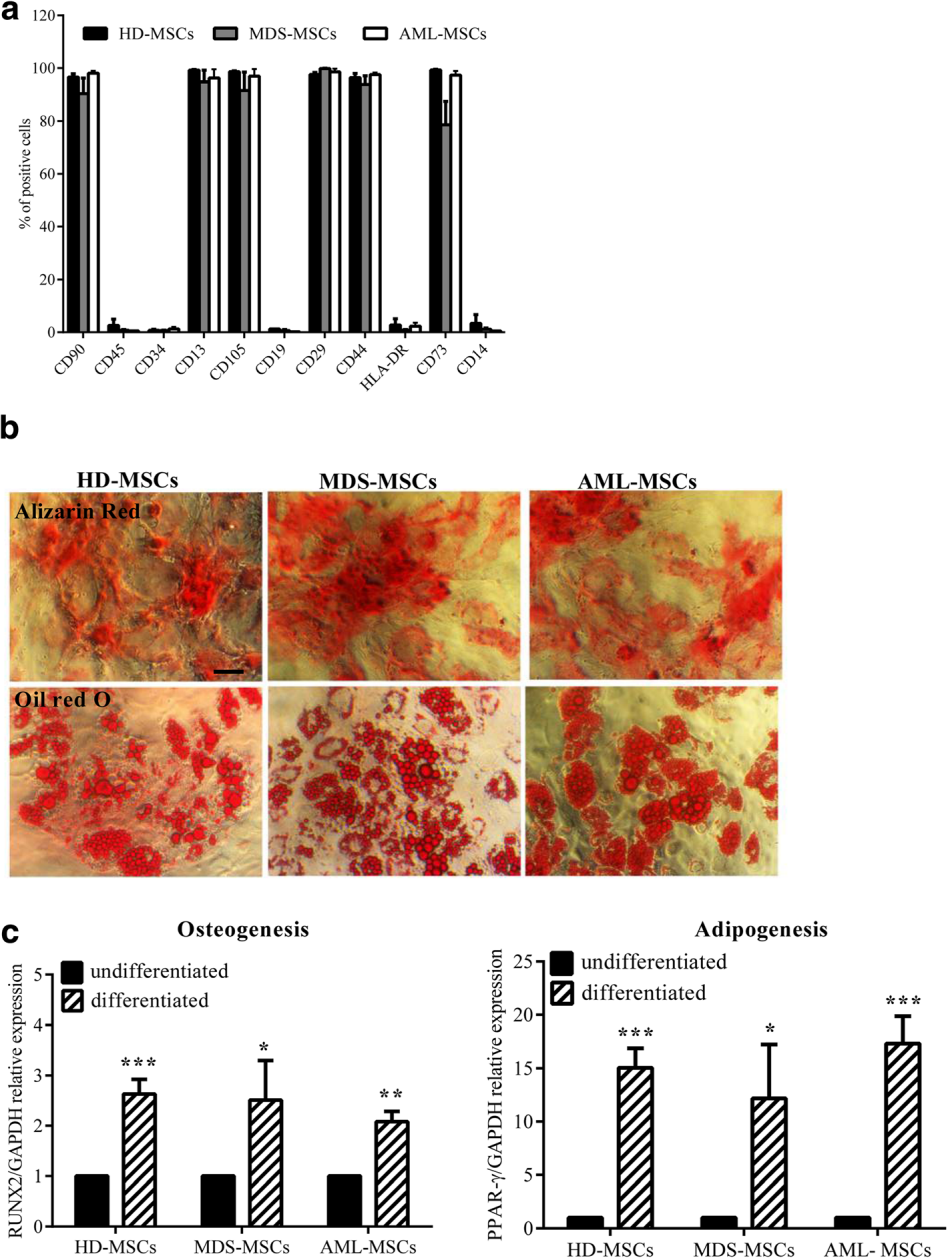
MDS-MSCs and AML-MSCs show typical MSC biological properties. **a** Flow cytometry analysis of HD-MSC (*N* = 9), MDS-MSC (*N* = 3), and AML-MSC (*N* = 4) immunophenotype. Histograms represent percentage of cells positive for CD90, CD45, CD34, CD13, CD105, CD19, CD29, CD44, CD73, CD14, and HLA-DR. All differences not significant. **b** Representative microphotographs of Alizarin red (upper row) and Oil Red O (lower row) staining of HD-MSCs, MDS-MSCs, and AML-MSCs cultured for 3 weeks in osteogenic and adipogenic conditions, respectively. Magnification 10×; scale bar, 100 μm. **c** qRT-PCR analysis of Runx2 (Runx2/GAPDH relative levels) and PPARγ (PPARγ/GAPDH relative levels) in undifferentiated (black histograms) and differentiated (crossed histograms) cells following 3 weeks of culture. Expression levels of differentiation specific genes in undifferentiated cells taken as 1 (mean ± SEM of at least four independent experiments). *P < 0.05; **P < 0.01; ***P < 0.001. HD-MSC mesenchymal stromal cell from healthy donor, MDS-MSC mesenchymal stromal cell from myelodysplastic syndrome patient, AML-MSC mesenchymal stromal cell from acute myeloid leukemia patient, PPARγ peroxisome proliferator activated receptor gamma, RUNX2 Runt-related transcription factor 2, GAPDH glyceraldehyde 3-phosphate dehydrogenase

MSCs isolated as already described were expanded and analyzed at P3 or P4 to ascertain typical biological properties according to the minimal criteria to define “bona fide” MSCs derived from BM [26]. The immunophenotype of MSCs was therefore first characterized by flow cytometry. We found that both MDS-MSCs and AML-MSCs expressed typical MSC markers and were negative for hematopoietic markers in a comparable way to HD-MSCs (Fig. 2a). Some variability existed in MDS-MSCs in the expression levels of CD105 and CD73, but these differences were not statistically significant (Fig. 2a). Thus, these data indicated that both MDS-MSCs and AML-MSCs showed the proper MSC immunophenotype.

Next, we examined the differentiation capacity of MSCs isolated from MDS and AML patients, in comparison with MSCs isolated from HDs. Thus, MSCs were induced to differentiate versus the osteogenic or adipogenic lineage, as described in Methods, and analyzed after 3 weeks by differentiation-specific histological staining. As shown in Fig. 2b, we observed a significant and specific Alizarin red (top) and Oil Red O (bottom) positive staining, respectively, in osteogenic-differentiated and adipogenic-differentiated MSC cultures. However, no significant differences were detected in the intensity of differentiation-specific staining in MDS-MSCs and AML-MSCs in comparison with the staining in HD-MSCs. Differentiation-specific staining was not observed in MSCs of the three groups cultured without differentiation-inducing agents (data not shown). To better quantify the differentiation efficiency, we quantitatively evaluated the expression of osteogenic and adipogenic pivotal transcription factors, before and after MSC differentiation induction, by quantitative real-time-PCR (qRT-PCR). We found that, as expected, RUNX2 and PPARγ expression levels were effectively increased in differentiated MSCs, but no significant differences in induction level were detected between the HD, AML, and MDS groups (Fig. 2c). We also evaluated the expression levels of differentiation master genes in MDS-MSCs and AML-MSCs under nondifferentiating culture conditions and did not find any significant differences (data not shown). These data demonstrate that MDS-MSCs and AML-MDSs presented a normal and comparable differentiation capacity.

### MDS-MSCs and AML-MSCs do not harbor tumor-specific cytogenetic abnormalities

**Fig. 3:**
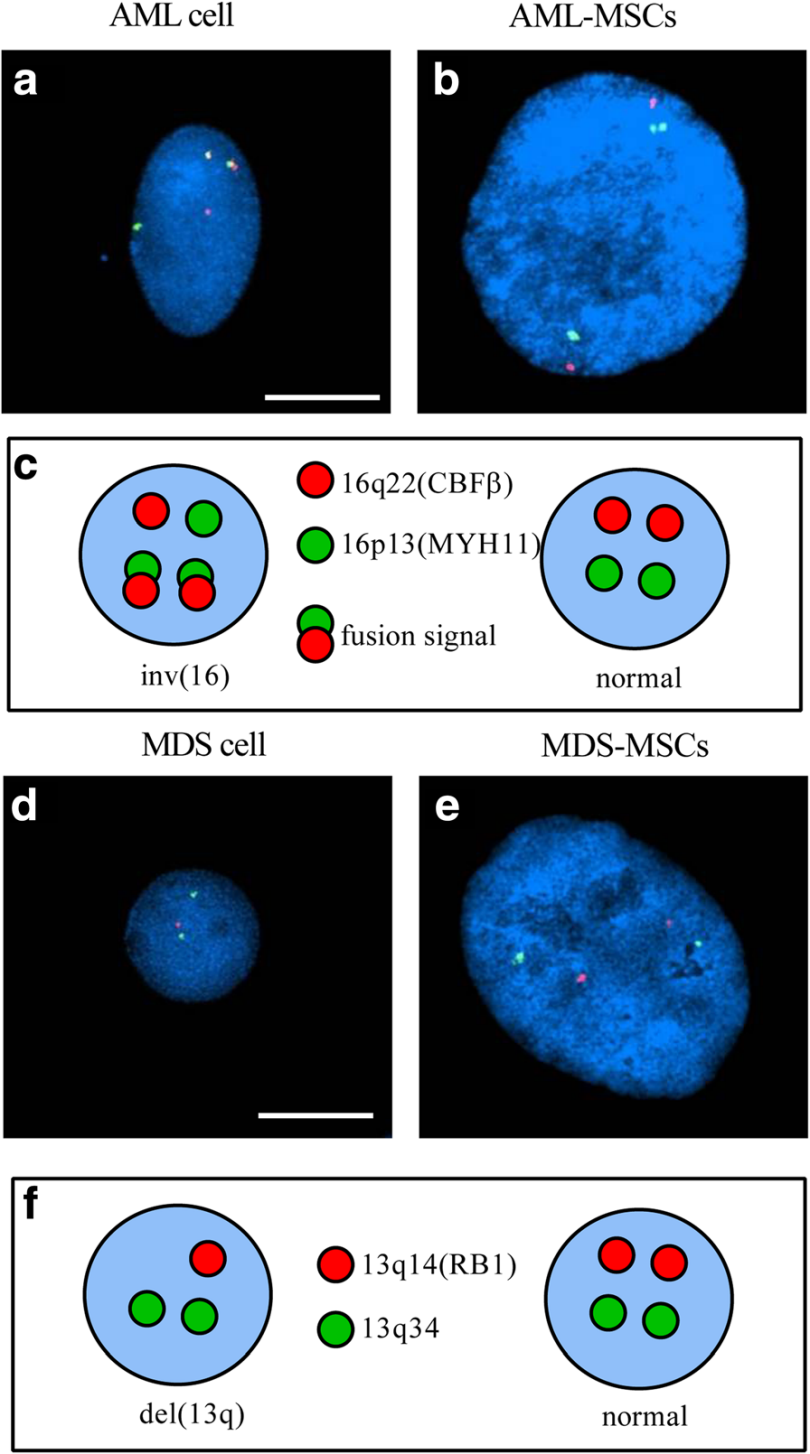
AML-MSCs and MDS-MSCs do not show tumor-specific chromosomal abnormalities. Representative samples analyzed by fluorescent in-situ hybridization (FISH). CBFβ/MYH11 Translocation, Dual Fusion Probe, and RB1 Deletion Probe used to investigate chromosome 16 (**a, b**) and chromosome 13 abnormalities (**c, d**), respectively. **a** BM-derived mononuclear cell with abnormal FISH pattern isolated from an AML patient. **b** MSC with normal FISH pattern isolated from same AML patient. **c** Schematic illustration of FISH probes and FISH signal pattern (**d**). BM-derived mononuclear cell with abnormal FISH pattern isolated from an MDS patient. **e** MSC with normal FISH pattern isolated from same MDS patient. **f** Schematic illustration of FISH probes and FISH signal pattern. Magnification (100×). Scale bar 10 μm. MDS-MSC mesenchymal stromal cell from myelodysplastic syndrome patient, AML-MSC mesenchymal stromal cell from acute myeloid leukemia patient

BM cells derived from hematological patients present, in most cases, tumor-specific genetic alterations. Thus, we decided to use FISH analysis to genetically characterize freshly isolated MNCs and MSCs, obtained as already described, in parallel from the BM of the same AML or MDS patient. As shown in Table 1, most of our patients (10 out of 24 AML patients and 16 out of 23 MDS patients) did not show cytogenetic defects in MNCs, so they were not suitable for FISH analysis on MSCs. In the analyzed samples (*N* = 3 MDS, *N* = 3 AML), we found that neither MDS-MSCs nor AML-MSCs presented the same chromosomal alterations, typical of myeloid malignancies, as those detected in MNCs at diagnosis (Fig. 3).

### MDS-MSCs and AML-MSCs equally support leukemic cell viability and proliferation

**Fig. 4:**
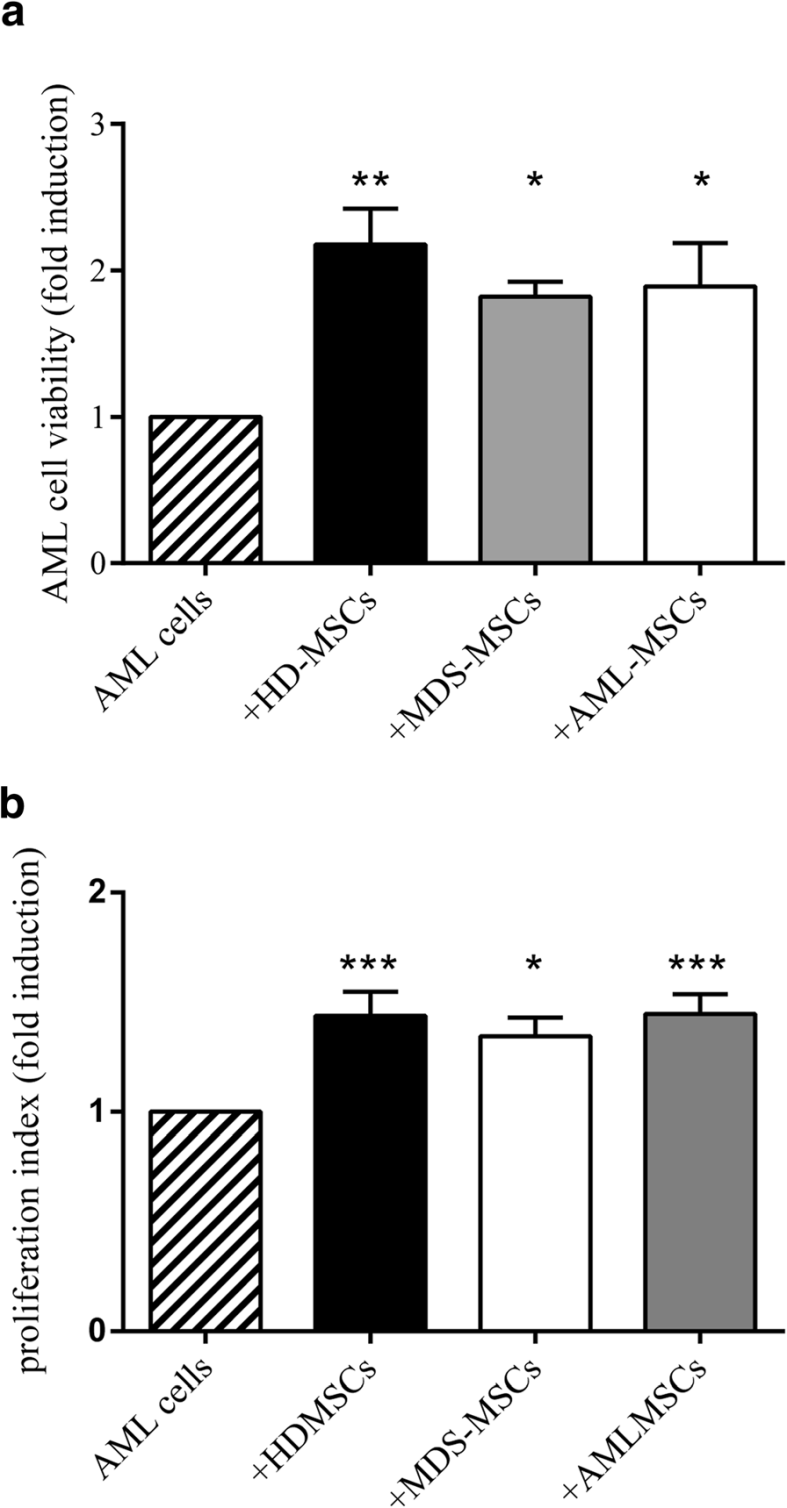
Cocultures with HD-MSCs, MDS-MSCs, and AML-MSCs increase AML cell viability and proliferation. **a** Viability rate established by evaluating double-negative cells in flow cytometer analysis of annexin/PI double-stained cells. Viability of AML cells cultured alone considered 1 (fold induction) (mean ± SEM of at least five independent experiments). *P < 0.05; ** *P* < 0.01 vs AML cells alone. Differences are not significant between MSC groups. **b** Cell proliferation of AML cells labeled with CFSE analyzed by flow cytometry. Proliferation rate evaluated taking proliferation of AML cell cultured alone as 1 (fold induction) (mean ± SEM of at least three independent experiments). *P < 0.05; ***P < 0.001 vs AML cells alone. Differences not significant between MSC groups. HD-MSC mesenchymal stromal cell from healthy donor, MDS-MSC mesenchymal stromal cell from myelodysplastic syndrome patient, AML-MSC mesenchymal stromal cell from acute myeloid leukemia patient

It has been shown that MSCs favor leukemic cell survival and inhibit apoptosis [27, 28]. We decided to test whether AML-MSCs and MDS-MSCs retained this capacity and if they showed differences among them. To rule out the bias due to the intrinsic variability between MDS-derived and AML-derived cells, AML cells isolated from the same AML samples were seeded onto irradiated HD-MSC, MDS-MSC, or AML-MSC layers and cocultured for 4 days, and then apoptosis was evaluated through annexin/PI staining by flow cytometry. We found that HD-MSCs, MDS-MSCs, and AML-MSCs significantly increased leukemic cell viability with no significant differences between groups (Fig. 4a).

To evaluate MSC capacity to stimulate AML cell proliferation, MSC/AML cell coculture experiments, similar to the one already described, were performed with CFSE-labeled AML cells. We found that HD-MSCs, MDS-MSCs, and AML-MSCs slightly but significantly stimulated AML cell proliferation No significant differences were found between groups (Fig. 4b).

### MDS-MSCs and AML-MSCs retain the ability to induce Tregs

**Fig. 5:**
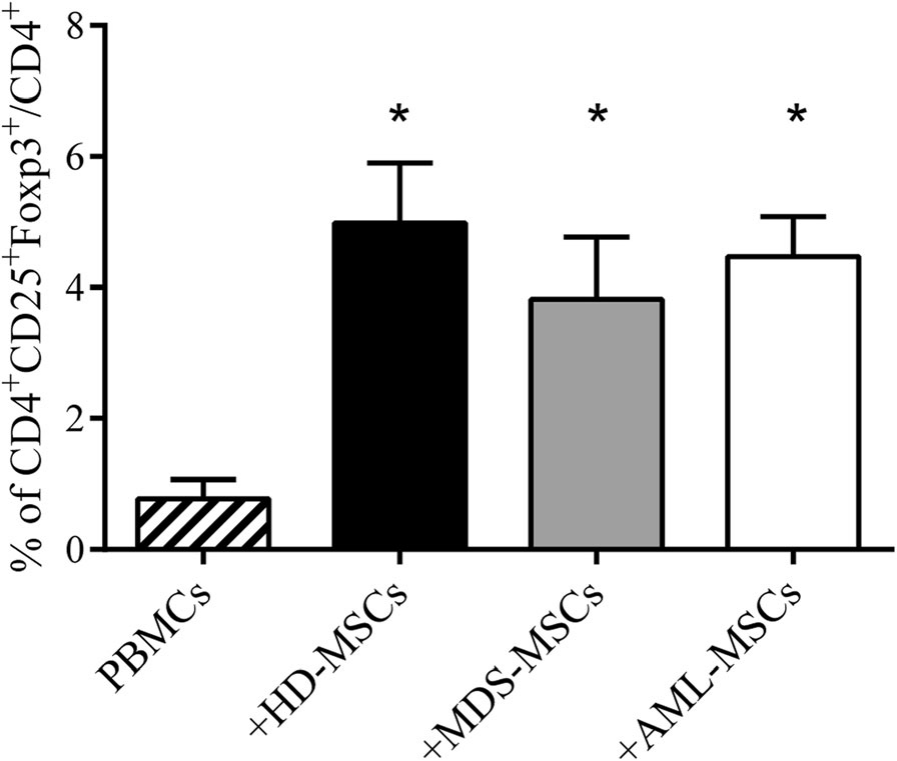
MDS-MSCs and AML-MSCs induce Tregs similar to HD-MSCs. Graph represents percentage of CD4^+^/CD25^+^/FoxP3^+^ cells analyzed by flow cytometry with respect to percentage of CD4^+^ cells. Tregs induced by PBMCs alone used as control (mean ± SEM of four independent experiments). *P < 0.05 vs PBMCs alone. Differences not significant between MSC groups. PBMC peripheral blood mononuclear cell, HD-MSC mesenchymal stromal cell from healthy donor, MDS-MSC mesenchymal stromal cell from myelodysplastic syndrome patient, AML-MSC mesenchymal stromal cell from acute myeloid leukemia patient

In AML, and especially in MDS, immune dysregulation participates in the establishment of a leukemic permissive milieu. We wondered whether there were differences in the immune-regulatory functions of HD-MSCs, MDS-MSCs, and AML-MSCs. In particular, we investigated the ability of MDS and AML patient-derived MSCs to induce CD4^+^/CD25^+^/FoxP3^+^ cells, that is, regulatory T cells (Tregs), which are known to suppress immunity also in hematological malignancies [29]. We therefore cocultured HD-MSCs, MDS-MSCs, and AML-MSCs with allogeneic PBMCs and, after 7 days, we evaluated the generated Tregs (i.e., CD4^+^/CD25^+^/FoxP3^+^ cells). We demonstrated that MDS-MSCs and AML-MSCs efficiently induced Tregs with no significant differences between them or versus HD-MSCs (Fig. 5). Thus, our data suggested that MDS-MSCs and AML-MSCs showed a comparable Treg-promoting activity.

## Discussion

In the last few years, the possible contribution of BM-MSCs to the pathogenetic/pathophysiologic process of MDS and AML has gained increasing interest. However, so far, understanding of the MSC role in supporting leukemia in vivo has been hindered by a low level of characterization and conflicting results. In particular, it has not been completely clarified whether AML-MSCs and MDS-MSCs share common features contributing to a disease-permissive microenvironment and preferentially supporting neoplastic cells.

In this study, we characterized and compared MSCs derived from MDS and AML patients with MSCs derived from HDs as a reference control. We showed that both MDS-derived and AML-derived MSCs met the MSC criteria proposed by the International Society for Cellular Therapy [26] and are similar in terms of phenotype and differentiation capacity. However, some functional differences can be underlined. We were unable to isolate MSCs from a substantial fraction of AML patients (25%, 8 out of 32), while almost all HD and MDS samples yielded MSCs. We did not find a correlation between this issue and a defined cytogenetic/molecular subgroup or patient age (see Table 1). We could hypothesize that a low number of precursors are present in the BM of these patients. Indeed, when we compared the frequency of rescued MSCs (i.e., the number of MSCs isolated at P1 normalized to the number of BM-isolated MNCs) we found that this ratio was significantly lower in the AML group than in the HD group, while MSC rescue in MDS has an intermediate value. We could therefore speculate that the reduced number of MSC precursors negatively impacted on MSC recovery and thus, below a given threshold, we were unable to isolate MSCs.

The main abnormality we detected in MDS-MSCs was a marked decrease in their proliferation potential. The growth pattern of MSCs isolated from MDS patients has been questioned, with some authors showing a reduced expansion potential [30–32], and others recording a proliferation rate similar to that of HD-MSCs [33–35]. We found that MDS-MSCs showed a proliferative capacity significantly lower in comparison to that of HD-MSCs. The proliferation rate of MDS-MSCs was also lower than AML-MSCs, albeit not significantly. Some authors showed that the growing defect in MSCs isolated from MDS patients was associated with cell senescence [36, 37]. However, we did not detect alterations in the MDS-MSC morphology, suggesting ongoing senescence. Rather, our unpublished data indicated that fetal bovine serum supplementation partially rescued MDS-MSC proliferation capacity, suggesting that MDS-MSCs displayed an intrinsic proliferation signaling defect, making them more dependent on growth factors.

It is noteworthy that, as expected, MDS samples in our study showed a higher median age than that of AML and HD samples. This could be relevant in differentiation assay results. Indeed, it was reported that MSC osteogenic and adipogenic potential decreased during aging [38–40]. However, we found that HD-MSC, MDS-MSC, and AML-MSC differentiation ability was maintained unchanged, regardless of the median age of the subjects. Indeed, we obtained similar results by comparing samples of similar age (data not shown). Some authors reported that MDS-derived MSCs displayed defective osteogenic and adipogenic lineage priming under nondifferentiating culture conditions [41]. However, when we evaluated the expression levels of differentiation master genes in MDS-MSCs and AML-MSCs under nondifferentiating culture conditions, we did not find significant differences (data not shown).

Some authors suggested that genetic alterations in MSCs might represent a specific mechanism of leukemogenesis. Indeed, they showed that MDS and AML patients, with genetic abnormalities in their in-vitro expanded MSCs, had a worse overall and disease-free survival than the normal karyotype [42]. However, others reported that, in spite of harboring severe chromosomal alterations, MSCs maintained normal functional properties [43]. The majority of patients analyzed in this study had a normal karyotype (see Table 1). Thus, FISH was performed in MDS and AML cases where an abnormality was found in neoplastic cells at diagnosis. In these cases, MSCs did not harbor the same cytogenetic abnormalities present in neoplastic cells. Although we could not rule out that MSCs presented genetic mutations different from their hematopoietic counterpart, we could conclude that, in our experience, neither MDS-MSCs nor AML-MSCs shared a common precursor with the original malignant clone. Our data are in agreement with a previous paper in which none of the 28 AML analyzed samples harbored tumor-specific cytogenetic alterations [19]*.* FISH data in MDS in the literature confirmed that cytogenetic aberrations in MDS-MSCs, if present, differed from chromosomal markers in altered hematopoietic cells [30, 33, 42].

MSCs have a unique immune-regulatory and immunosuppressive ability. Since aberrant immune responses have been associated with the pathophysiology of AML, and especially of MDS, we decided to test the immune-modulatory properties of MDS-MSCs and AML-MSCs. In particular, we decided to investigate the ability of MDS and AML patient-derived MSCs to induce Tregs. Indeed, Tregs have been recognized as essential contributors in microenvironment immunomodulation and ultimately in helping leukemic cells to evade immune surveillance [29, 44]. In the tumor microenvironment, Tregs interact with diverse cell subsets, including MSCs, able to enhance their suppressive function [45, 46]. Previous studies on this issue are limited and controversial [32, 47, 48]. Our data showed that MDS-MSCs and AML-MSCs were able to induce Tregs with an efficiency comparable to that of HD-MSCs. Moreover, our unpublished results indicate that, like HD-MSCs, MDS-MSCs and AML-MSCs expressed low basal levels of indoleamine 2,3 deoxygenase (IDO)-1 enzyme, which plays an important role in Treg modulation. MDS-MSCs and AML-MSCs also upregulated IDO1 following proinflammatory cytokine treatment to a similar extent with respect to HD-MSCs (our unpublished data). Thus, our data suggest that MDS-MSCs and AML MSCs showed comparable immunoregulatory functions.

It has been demonstrated that MDS-MSCs are defective in hematopoiesis supporting functions [37, 49, 50], while conflicting results have been obtained in AML-MSCs [18, 51, 52]. Murine transplant experiments elegantly demonstrated that neoplastic cells shared the BM milieu with their nonneoplastic counterpart, so that leukemic cells competed with normal HSCs for occupancy of the same protective niche [52]. In this paper, we demonstrated that MDS-MSCs supported AML cell viability and proliferation in vitro as well as HD-MSCs and AML-MSCs. Since MSC aging—which, as expected, occurs in most MDS cases—negatively impacted on hematopoiesis, we could speculate that the MSC capacity to sustain leukemic cell viability and proliferation in vitro together with the impaired hematopoiesis-supporting ability, particularly in MDS–MSCs, could virtually contribute to favor a disease-permissive niche.

## Conclusions

Our data demonstrate that MDS-MSCs and AML-MSCs share common features such as phenotype, differentiation capacity, absence of leukemia-specific genetic abnormalities, ability to sustain AML cell viability, and immune-regulatory capacity in vitro. However, AML-MSCs were more difficult to isolate from BM, while MDS-MSCs showed a lower proliferation potential. Overall, MDS-MSCs and AML-MSCs did not present macroscopic defects and/or abnormalities directly related to leukemia, but both displayed functional differences that, translated in vivo, could potentially help to turn the BM microenvironment from hostile to supportive for leukemic cells.

## Abbreviations

AML: Acute myeloid leukemia
AML-MSC: MSC isolated from acute myeloid leukemia patients
BM: Bone marrow
CFSE: Carboxyfluorescein succinimidyl ester
DMEM: Dulbecco’s modified Eagle’s medium
FBS: Fetal bovine serum
FISH: Fluorescence in situ hybridization
FITC: Fluorescein isothiocyanate
GAPDH: Glyceraldehyde 3-phosphate dehydrogenase
HD: Healthy donor
HD-MSC: MSC isolated from healthy donors
HSC: Hematopoietic stem cell
MCGS: Mesenchymal cell growth serum
MDS: Myelodysplastic syndrome
MDS-MSC: MSC isolated from myelodysplastic syndrome patients
MNC: Mononuclear seeded cell
MSC: Mesenchymal stromal cell
PBMC: Peripheral blood mononuclear cell
PBS: Phosphate-buffered saline solution
PE: Phycoerythrin
PFA: Paraformaldehyde
PPARγ: Peroxisome proliferator activated receptor gamma
qRT-PCR: Quantitative real-time polymerase chain reaction
RUNX2: Runt-related transcription factor 2
Treg: Regulatory T cell
